# Pneumatosis intestinalis with portal, mesenteric and renal gas due to colonic pseudo-obstruction

**DOI:** 10.1515/iss-2021-0031

**Published:** 2022-06-28

**Authors:** Eliane Dohner, Marc von Tobel, Samuel Käser, René Fahrner

**Affiliations:** Department of Surgery, Bürgerspital Solothurn, Solothurn, Switzerland; Department of Anaesthesiology and Intensive Care Medicine, Bürgerspital Solothurn, Solothurn, Switzerland

**Keywords:** colonic pseudo-obstruction, mesenteric ischemia, pneumatosis intestinalis, portal gas

## Abstract

**Objectives:**

Pneumatosis intestinalis is a rare condition with subserosal or submucosal gas-filled cysts of the gastrointestinal tract. It is often associated with acute mesenteric ischemia, but also non-ischemic causes are described.

**Case presentation:**

A 27-year-old male patient with severe congenital spastic tetraparesis presented to the emergency room with fever and reduced general condition. The patient was hypotonic and tachycardic, had a fever up to 39.7 °C and reduced peripheral oxygen saturation. The laboratory analyses revealed leukocytosis (16.7 G/L) and elevated CRP (162 mg/L).

The patient was admitted to the intensive care unit (ICU) for invasive ventilator treatment because of global respiratory insufficiency and antibiotic therapy due to acute pneumonia and severe acute respiratory distress syndrome (ARDS). In addition, he suffered from colonic pseudo-obstruction but with persistent stool passage. After pulmonary recovery, he was transferred to the normal ward of internal medicine, but signs of colonic pseudo-obstruction were still present.

Under therapy with diatrizoic acid and neostigmine, the abdomen was less distended, and the patient had regular bowel movements. After four days, the patient developed sudden acute abdominal pain and suffered sudden pulseless electrical activity. Immediate cardiopulmonary resuscitation was provided. After the return of spontaneous circulation, the patient underwent computed tomography (CT) and was re-admitted to the ICU. The CT scan showed massive dilatation of the colon, including pneumatosis coli, extensive gas formation within the mesenteric veins and arteries, including massive portal gas in the liver, the splenic vein, the renal veins, and disruption of abdominal aortic perfusion. The patient was then first presented for surgical evaluation, but due to futile prognosis, treatment was ceased on the ICU.

**Conclusions:**

In conclusion, colonic pseudo-obstruction might have led to colonic necrosis and consecutive massive gas formation within the mesenteric vessels. Therefore, intestinal passage should be restored as soon as possible to avoid possible mortality.

## Introduction

Pneumatosis intestinalis is a rare condition with subserosal or submucosal gas-filled cysts of the gastrointestinal tract, which may be detected in radiographs or computer tomography (CT) scans. It is most often associated with acute mesenteric ischemia, but cases of nonischemic causes such as chronic obstructive pulmonary disease, leukemia, intake of steroids, chemotherapy, AIDS, connective tissue disorders, celiac disease, or infectious enteritis have also been described [1]. In association with portomesenteric venous gas, pneumatosis intestinalis is often a radiological sign of bowel ischemia and is associated with a high mortality rate of up to 75% [2]. However, overall, these signs do not point to specific diagnoses but are general symptoms or radiological signs, which have several medical etiologies [3].

Colonic pseudo-obstruction was first described in 1948 by Sir Ogilvie [4], who described massive colonic dilatation without a distinguishable mechanical obstruction [5]. The definite mechanism is still under investigation, and various hypotheses exist. Several conditions are associated with the occurrence of acute colonic pseudo-obstruction, including cardiovascular disease, metabolic changes, drug-induced, infectious or inflammatory states, neurological diseases, neoplasia, and posttraumatic or postoperative states [5]. In severe cases, colonic pseudo-obstruction may lead to perforation or ischemia and is then associated with a high mortality rate [5]. Treatment options range from medical treatment, endoscopic interventions, or, in case of treatment failure, surgery [5, 6].

We report here a case of a young patient with chronic colonic pseudo-obstruction with a fatal outcome due to cardiac arrest and pneumatosis intestinalis. In addition, gas was detected in the portomesenteric veins, renal vein, mesenteric artery and acute occlusion of the abdominal aorta.

## Case report

**Figure 1:**
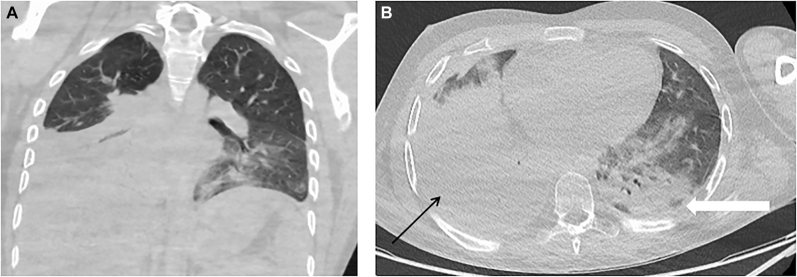
CT scan of the thorax upon admission showing signs of pleural effusion (thin black arrow) and inflammatory infiltrations (bold white arrow) which lead to global respiratory insufficiency.

**Figure 2:**
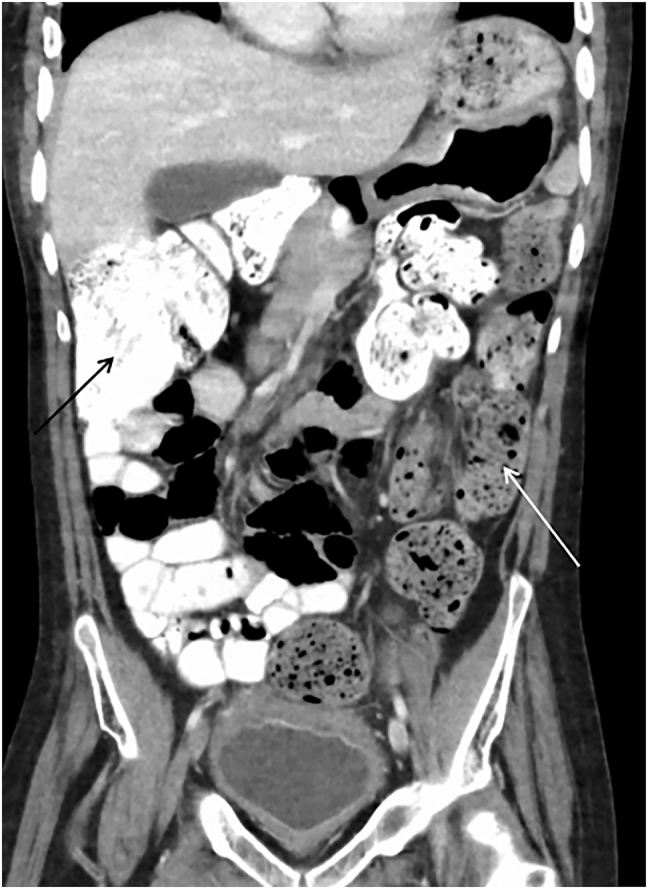
CT scan of the abdomen two years prior to the acute presentation, showing chronic dilatated and congested colon (thin arrows) without obvious mechanical obstruction, yet the presence of colonic dilatation up to 8 cm. Orally administered contrast medium was detected within the small intestine and ascending to the transverse colon but not in the descending colon.

A 27-year-old male patient with congenital severe spastic tetraparesis presented to the emergency room with fever and reduced general condition after a dental intervention with general anesthesia days before. The patient was hypotonic and tachycardic and had fever up to 39.7 °C, and peripheral oxygen saturation was reduced. The laboratory analyses revealed leukocytosis (16.7 G/L) and an elevated CRP (162 mg/L). Because of global respiratory insufficiency, the patient was admitted to the intensive care unit (ICU) for invasive ventilator treatment and antibiotic therapy due to acute pneumonia and ARDS (Figure 1A, B). Prior to admission, he endured years of chronic colonic pseudo-obstruction and constipation but with persistent stool passage (Figure 2). After pulmonary recovery, he was transferred to the normal internal medicine ward for further pulmonary resuscitation and stool passage optimization due to colonic pseudo-obstruction.

**Figure 3:**
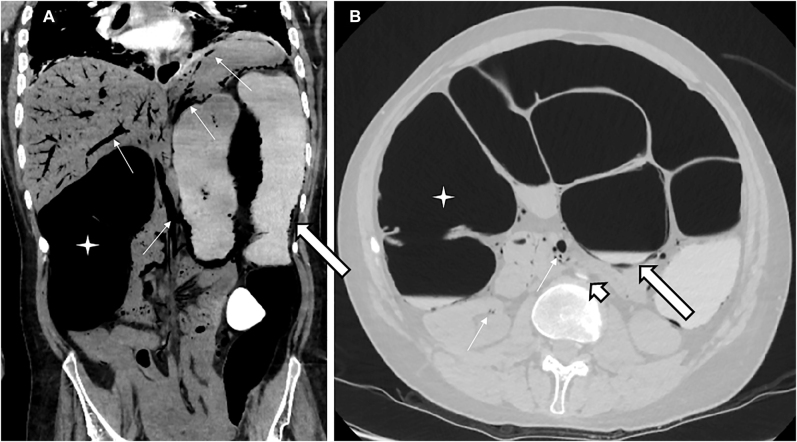
A CT scan of the abdomen showed massive dilatation of the colon (star), including pneumatosis coli (long bold arrows), extensive gas formation (thin arrows) within the mesenteric veins and arteries, including massive amounts of portal gas in the entire portal basin of the liver, splenic vein, renal veins, and the abdominal aorta, leading to disruption of perfusion (short bold arrow).

Under therapy with diatrizoic acid and neostigmine, the abdomen was less distended, and the patient had regular bowel movements with daily stool passage. Hence, no endoscopic intervention was performed. After four days, the patient developed sudden acute abdominal pain and experienced sudden pulseless electrical activity. Immediate cardiopulmonary resuscitation was provided. After the return of spontaneous circulation was obtained, the patient underwent CT and was referred to the ICU again. The CT scan showed massive dilatation of the colon, including pneumatosis coli; extensive gas formation within the mesenteric veins and arteries, including massive amounts of portal gas in the entire portal basin of the liver, splenic vein, and renal veins; and disruption of abdominal aortic perfusion (Figure 3A, B). For further treatment evaluation, the patient was first assessed for possible surgical exploration, but due to poor prognosis (high catecholamine doses, pH 7.01, lactate 14.6 mmol/l), treatment was ceased in the ICU with consent from the next of kin.

## Discussion

We report a fatal course of colonic pseudo-obstruction in a young patient, who developed cardiac arrest with consecutive extensive gas formation within the portal, mesenteric, and renal vessels, leading to eventual compression of the aorta.

Due to congenital spastic tetraparesis, the patient had restricted movement since his birth; therefore, therapeutic and surgical interventions were approached in a conservative manner, as the family preferred a limited approach to surgical interventions. For example, a planned cholecystectomy was delayed as the severity of symptoms was, from the perspective of the family, not sufficient to perform an operation under general anesthesia. Therefore, intermittent and chronic colonic pseudo-obstruction was treated conservatively for many years.

In the case of uncomplicated colonic pseudo-obstruction, a conservative treatment including fasting, nasogastric tube insertion, fluids and discontinuation of medication impacting colonic motility, might be performed for approximately 72 h. If conservative treatment fails or the cecal diameter dilates more than 12 cm, administration of short-acting anticholinesterase medication can stimulate colonic motility. In failure of this treatment, endoscopic decompression with a tube placed in the cecum is a viable option, and in cases with complications or signs of ischemia or perforation, surgery remains the last treatment option [7]. In this specific case, stool passage was present under conservative treatment, so endoscopic or surgical decompression was not considered.

The exact mechanism of this fatal course, following dental intervention under general anesthesia which had taken place a few days earlier, remains unclear and is complex. We suspected a potential aspiration with consecutive pneumonia and global respiratory insufficiency leading to ARDS and ICU therapy. This pulmonary deterioration worsened arterial perfusion of oxygen within the intestinal branches and promoted a potential oxygen shortage within the mucosal layer, leading to gradual necrosis and deterioration of the intestinal passage with increasing bowel distention. Subsequently, despite temporary clinical recovery, the patient developed an enlarged abdominal compartment with a further increase in abdominal pressure, reducing intestinal arterial perfusion and increasing intestinal ischemia, leading to momentary cardiac arrest.

The observed massive radiological alterations with pneumatosis intestinalis, gas formation within the portal basin including the liver, splenic vein, mesenteric and renal veins, and even disruption of abdominal aortic perfusion were, in this case, late and fatal signs of intestinal ischemia.

In conclusion, pre-existing colonic pseudo-obstruction might have led to a fatal deterioration of bowel perfusion with gradual intestinal necrosis and massive gas formation within the venous and arterial mesenteric vessels. In addition, an abdominal compartment syndrome can be assumed with further deterioration of the intestinal perfusion. This case demonstrates that restoration of the intestinal passage in colonic pseudo-obstruction should be obtained as soon as possible, and/or early and regular evaluation by a surgeon is necessary to avoid possible fatal courses.

## Supplementary Material

Supplementary Material
