# Supplementary material for: Pneumatosis intestinalis with portal, mesenteric and renal gas due to colonic pseudo-obstruction
Source: Innov Surg Sci. 2022 Jun 28;7(1):31–4. doi: 10.1515/iss-2021-0031 (PMC9352184; doi:10.1515/iss-2021-0031)
Supplement: Supplementary file 1 — Supplementary Material [file j_iss-2021-0031_suppl.pdf]

Reviewer Assessment

R. Fahrner et al.: Pneumatosis intestinalis with portal, mesenteric and renal gas due to colonic pseudo-obstruction

Reviewers’ Comments to Original Submission

Reviewer 1: Tuchmann, Albert

Date received: 28-Sep-2021
Reviewer recommendation: **Return to author for major modifications**
Reviewer overall scoring: **High**

Assessment Form scores: 5 = High/Yes; 3 = Medium/Adequate; 1 = Low

Is the subject area appropriate for the journal		4			
Does the title clearly reflect the paper's content?		4			
Does the abstract clearly reflect the paper's content	5				
Do the keywords clearly reflect the paper's content?	5				
Does the introduction present the problem clearly?		4			
Are the results/ conclusions justified?			3		
How comprehensive and up-to-date is the subject matter presented?				2	
How adequate is the data presentation?			3		
Are units and terminology used correctly?		4			
Is the number of cases adequate?			3		
Are the experimental methods/ clinical studies adequate?				2	
Is the length appropriate in relation to the content?				2	
Does the reader get new insights from the article?		4			
Please rate the practical significance.		4			
Please rate the accuracy of methods.			3		
Please rate the statistical evaluation and quality control.					
Please rate the appropriateness of the figures and tables.		4			
Please rate the appropriateness of the references.				2	
Please evaluate the writing style and use of language.					1
Please judge the overall scientific quality of the manuscript.				2	
Are the methods used worthy of reproduction in greater deal?	Yes				
Would you be willing to review a revision of this manuscript?	Yes				

Comments to author: Bitte sprachlich überarbeiten, am besten durch native speaker!. Die Abbildungen besser erklären, z.B. durch Pfeile bei allen CT Bildern.

Der deutsche Titel der Arbeit kann so nicht stimmen, ist nicht "deutsch".

Reviewer 2: Ulrich, Alexis

Date received: 07-Nov-2021
Reviewer recommendation: **Return to author for minor modifications**
Reviewer overall scoring: **Medium**

Assessment Form scores: 5 = High/Yes; 3 = Medium/Adequate; 1 = Low

Is the subject area appropriate for the journal		4			
Does the title clearly reflect the paper's content?		4			
Does the abstract clearly reflect the paper's content		4			
Do the keywords clearly reflect the paper's content?		4			
Does the introduction present the problem clearly?		4			
Are the results/ conclusions justified?		4			
How comprehensive and up-to-date is the subject matter presented?		4			
How adequate is the data presentation?			3		
Are units and terminology used correctly?		4			
Is the number of cases adequate?		4			
Are the experimental methods/ clinical studies adequate?					
Is the length appropriate in relation to the content?		4			
Does the reader get new insights from the article?			3		
Please rate the practical significance.			3		
Please rate the accuracy of methods.			3		
Please rate the statistical evaluation and quality control.			3		
Please rate the appropriateness of the figures and tables.		4			
Please rate the appropriateness of the references.		4			
Please evaluate the writing style and use of language.		4			
Please judge the overall scientific quality of the manuscript.			3		
Are the methods used worthy of reproduction in greater deal?			No		
Would you be willing to review a revision of this manuscript?			Yes		

Comments to author: The authors present a case report about a young patient with pneumatosis intestinal after chronic colonic pseudo obstruction.
The paper is well written, however there are a few minor comments:
1. The conclusion was, that the obstruction would have better been treated earlier. Please define what treatment would have been appropriate at which time.
2. was an endoscopic relief tried after detection of the pseudo obstruction?

Authors' Response to Reviewer Comments

Date received: 08-Jan-2022

Response to reviewer 1

Comments to the Author

Bitte sprachlich überarbeiten, am besten durch native speaker!

Wir haben das Manuskript durch ein professionelles Sprachediting überarbeiten lassen – siehe Zertifikat.

Die Abbildungen besser erklären, z.B. durch Pfeile bei allen CT Bildern.

Zur besseren Verständnis der Abbildungen, haben wir weitere Pfeile eingefügt und die Abbildungen in den Legenden noch besser beschrieben und erklärt.

Der deutsche Titel der Arbeit kann so nicht stimmen, ist nicht "deutsch".

Vielen Dank für den Kommentar. Wir haben den Titel auf der Titelseite entsprechend geändert.

Response to reviewer 2

Comments to the Author

The conclusion was, that the obstruction would have better been treated earlier. Please define what treatment would have been appropriate at which time.

We included a short section of proposed treatment in a recently published review by Conner et al (page 5, third paragraph).

Was an endoscopic relief tried after detection of the pseudo obstruction?

As the patient still had stool passage under conservative and medical treatment, there was no endoscopic intervention performed. In the acute phase of deterioration, the was no indication for endoscopic treatment (page 4, 2nd paragraph).

Reviewers' Comments to the Revised Manuscript

Reviewer 1: Tuchmann, Albert

Date received: 26-Jan-2022
Reviewer recommendation: **Accept in present form**
Reviewer overall scoring: **Medium**

Assessment Form scores: 5 = High/Yes; 3 = Medium/Adequate; 1 = Low

Is the subject area appropriate for the journal			3		
Does the title clearly reflect the paper's content?	5				
Does the abstract clearly reflect the paper's content	5				
Do the keywords clearly reflect the paper's content?	5				
Does the introduction present the problem clearly?	5				
Are the results/ conclusions justified?			3		

How comprehensive and up-to-date is the subject matter presented?		4			
How adequate is the data presentation?		4			
Are units and terminology used correctly?		4			
Is the number of cases adequate?			3		
Are the experimental methods/ clinical studies adequate?				2	
Is the length appropriate in relation to the content?		4			
Does the reader get new insights from the article?		4			
Please rate the practical significance.			3		
Please rate the accuracy of methods.		4			
Please rate the statistical evaluation and quality control.				2	
Please rate the appropriateness of the figures and tables.			3		
Please rate the appropriateness of the references.			3		
Please evaluate the writing style and use of language.		4			
Please judge the overall scientific quality of the manuscript.			3		
Are the methods used worthy of reproduction in greater deal?	Yes				
Would you be willing to review a revision of this manuscript?	Yes				

Comments to author: none. - One case, readers can learn something..

Reviewer 2: Ulrich, Alexis

Date received: 29-Jan-2022

Reviewer recommendation: **Accept in present form**

Reviewer overall scoring: **High**

Assessment Form scores: 5 = High/Yes; 3 = Medium/Adequate; 1 = Low

Is the subject area appropriate for the journal		4			
Does the title clearly reflect the paper's content?		4			
Does the abstract clearly reflect the paper's content		4			
Do the keywords clearly reflect the paper's content?		4			
Does the introduction present the problem clearly?		4			
Are the results/ conclusions justified?		4			
How comprehensive and up-to-date is the subject matter presented?		4			
How adequate is the data presentation?		4			
Are units and terminology used correctly?		4			
Is the number of cases adequate?		4			
Are the experimental methods/ clinical studies adequate?		4			
Is the length appropriate in relation to the content?		4			
Does the reader get new insights from the article?			3		
Please rate the practical significance.			3		
Please rate the accuracy of methods.		4			

Please rate the statistical evaluation and quality control.			3		
Please rate the appropriateness of the figures and tables.		4			
Please rate the appropriateness of the references.		4			
Please evaluate the writing style and use of language.		4			
Please judge the overall scientific quality of the manuscript.			3		
Are the methods used worthy of reproduction in greater deal?					
Would you be willing to review a revision of this manuscript?	Yes				

Comments to author: The reviewers suggestions were addressed sufficiently.

Comments by the Editor-in-Chief to Revised Submission

All reviewer comments were addressed adequately and the manuscript should be published in its present stage.
